# Estrogen-Regulated Proline-Rich Acidic Protein 1 in Endometrial Epithelial Cells Affects Embryo Implantation by Regulating Mucin 1 in Mice

**DOI:** 10.3390/biom15060852

**Published:** 2025-06-11

**Authors:** Xueyan Wang, Meng Li, Jingmei Han, Nana Yang, Xiangyun Li, Xinglong Wu

**Affiliations:** 1 College of Animal Science and Technology, Hebei Agricultural University, No. 289 Lingyusi Street, Baoding 071001, China; wangxueyan722@163.com (X.W.); limengtudou@163.com (M.L.); 17693378189@163.com (N.Y.); lxyun@hebau.edu.cn (X.L.); 2 College of Veterinary Medicine, Hebei Agricultural University, Baoding 071001, China; hanjingmei0717@163.com

**Keywords:** proline-rich acidic protein 1, embryo adhesion, estrogen, mucin 1, O-glycosylation

## Abstract

This study explores the regulatory role of proline-rich acidic protein 1 (*Prap1*) effects on embryo implantation. A high estrogen model and ovariectomized mice were employed to demonstrate that estrogen regulates *Prap1* expression. Uterine tissues were collected from E 1.5 (the presence of a vaginal plug was recorded as embryonal day 0.5, E 0.5) to E 7.5 to detect the *Prap1* expression pattern in early pregnancy using qRT-PCR. Embryo adhesion was assessed through uterine perfusion of PRAP1 protein and *Prap1* overexpression in endometrial epithelial cells (EECs). The data showed that *Prap1* expression was increased in the uterus with high estrogen levels. *Prap1* expression was specifically reduced during early implantation. Overexpression of *Prap1* in EECs also reduced the embryo adhesions. The differentially expressed genes obtained by RNA-seq were enriched in extracellular matrix and cell adhesion. *Muc1* expression was increased in EECs overexpressing *Prap1* by RNA-seq and qRT-PCR. Similarly, O-glycosylation biosynthesis was enriched, and glycosylation-related genes were upregulated. Our results demonstrate that *Prap1* was regulated by estrogen and an increase in PRAP1 before implantation affected embryo adhesion by regulating the expression of *Muc1* and extracellular matrix-related genes, leading to embryo implantation failure. Our results provide a new insight into estrogen regulation of embryo implantation.

## 1. Introduction

During embryo implantation, the endometrial epithelial cell (EEC) is the first cell type to contact the embryo, establishing maternal–fetal interaction [1]. These cells undergo a series of cellular and molecular changes, including loss of apical microvilli, alterations in apical adhesiveness, changes in the basal lateral membrane, modifications in intracellular vesicle transport, and immune tolerance development, to establish the transient uterine receptivity for embryo implantation. This process is primarily regulated by ovarian hormones estrogen (E_2_) and progesterone (P_4_), enabling the endometrium to become receptive to the embryo [2,3]. The implantation window is the receptive period during which the uterus allows the embryo to implant and typically occurs E 4–E 4.5 days in mice (the presence of a vaginal plug was recorded as embryonal day 0.5, E 0.5) [4]. A low estrogen level determines the opening of the implantation window, while a high estrogen level can prematurely close it by causing the abnormal expression of implantation-related genes and impairing endometrial receptivity [5,6]. Abnormally high estrogen levels in humans can lead to pregnancy disorders [7,8]. Controlled ovarian hyperstimulation (COH) commonly increases estrogen levels in intensive livestock production and human-assisted reproductive technology. Research investigating the impact of COH-induced estrogen levels on embryo implantation suggests that high estrogen levels may negatively affect endometrial remodeling [9]. High estrogen levels prevented the transformation of the luminal epithelium from a non-adherent to an adherent barrier, potentially altering endometrial receptivity and causing premature closure of the implantation window [9,10]. Studies have reported elevated estrogen levels due to ovarian stimulation with implantation failure, as they can make the endometrium less receptive or non-receptive [5,11]. These findings suggested the detrimental impact of supraphysiological estrogen levels on endometrial receptivity.

Our previous study revealed that a supraphysiological estrogen level produced during controlled ovarian stimulation (COS) could result in embryo implantation failure, accompanied by a significantly increased mRNA expression of proline-rich acidic protein 1 (*Prap1*) [12]. PRAP1 is a 17 kDa-secreted protein composed of 149 amino acids found in epithelial cells of the gastrointestinal tract, uterus, liver, and kidneys [13,14,15,16]. It plays a role in cancer responses by affecting the cell cycle, cell migration ability, and immune microenvironment, thereby maintaining epithelial cell homeostasis [15,17,18]. As a cell protective factor, PRAP1 is also involved in multiple cellular damage responses, cellular stress, and repair-oriented targets [19,20]. PRAP1 is abundantly expressed in the uterus in late pregnancy and disappears within 3 days after birth, previously known as pregnancy-specific uterine protein (PSUP) [21,22]. Furthermore, PRAP1 expression progressively rises during decidualization, and neutralizing PRAP1 can reduce the number of embryo implantation sites [13]. Our previous study found that *Prap1* mRNA expression was higher in the uteri of mice with superphysiological estrogen levels on E 3.5 [12]. We hypothesized that abnormally high *Prap1* expression is associated with reduced embryo implantation rate.

Therefore, the present study investigated the regulatory relationship between estrogen and *Prap1*, as well as the mechanism of *Prap1* to regulate embryo implantation. This study provides new insights into the mechanisms by which high estrogen affects embryo implantation.

## 2. Materials and Methods

### 2.1. Animals

CD-1 mice aged 6–8 weeks were purchased from the Vital River Laboratory Animal Technology Co., Ltd. (Beijing, China). All mice were housed under controlled conditions (12 h light/12 h dark, temperature: 22–24 °C, Humidity: 55–65%) and free access to food and water. The Animal Use and Care Committee of Hebei Agricultural University approved all animal experiments.

### 2.2. Treatments and Samples Collection

Female mice were intraperitoneally injected with 7.5 IU of pregnant mare serum gonadotropin (PMSG, Ningbo Sansheng, Zhejiang, China), followed by an intraperitoneal injection of 3.5 μg of gonadotropin-releasing hormone (GnRH, Selleckchem, Shanghai, China) 48 h later in the superovulation group (PG). Meanwhile, these mice and natural estrus female mice (Control group, Ctr) were mated with fertile males. The presence of a vaginal plug the next morning was recorded as embryonal day 0.5 (E 0.5). Blood samples were collected from the PG and Ctr group mice on E 3.5 to separate serum. Blood was collected from the orbital venous plexus into 200 μL PCR tubes. The blood was allowed to stand at 4 °C for 6 h and then centrifuged at 3000× *g*/min for 10 min. The serum was aspirated and stored at −20 °C for later use. Uterine tissue was collected for the PG group on E 3.5 and the natural pregnancy group on E 1.5–E 7.5 and stored at −80 °C for further studies.

Sesame oil (0.1 mL/mouse), estradiol-17β (300 ng/0.1 mL/mouse), and estradiol 17β combined with estrogen antagonist ICI 182780 (300 ng/0.1 mL/mouse) were subcutaneously injected into the neck of ovariectomized mice. After 24 h, the uterus was collected and stored at −80 °C.

The uterine horn of mice was surgically exposed, followed by the infusion of 3 μL of PRAP1 protein (50 μg/mL, Novoprotein, Shanghai, China) into the uterine cavity using a mouth pipette at the uterotubal junction on E 2.5. The contralateral uterine horn received an equivalent volume of saline via the same method. Implantation sites were assessed by observing decidualization on E 7.5.

### 2.3. Enzyme-Linked Immunosorbent Assay (ELISA)

Estrogen levels using an E_2_ enzyme-linked immunosorbent assay (ELISA) kit (Shanghai Jianglai Biotechnology Co., Ltd., Shanghai, China) for analysis. The method of testing serum samples was performed according to the manufacturer’s instructions. The ELISA kit on the intra-assay CV was less than 9%, the inter-assay CV was less than 11%, and the assay sensitivity was 0.1 pg/mL.

### 2.4. Primary Mouse EECs Culture

The uterus of estrus female mice was taken and rinsed well with DPBS containing penicillin–streptomycin. Subsequently, the endometrium was mechanically dissected using sharp forceps under a microscope and placed in a mixture solution of collagenase II (2 mg/mL), collagenase IV (2 mg/mL), and DNase I (50 μg/mL), followed by digested on a constant temperature shaker (37 °C, 600 rpm) for 30 min. The digested cell suspension with a 100 μm mesh filter and the filtrate were collected. Then, the filtrate was centrifuged at 600 rpm for 5 min, and the supernatant was discarded. The cell pellet was resuspended in a complete medium. Stromal cells were removed by differential adherence. The EESs were seeded in a culture plate and placed in an incubator at 37 °C with 5% CO_2_ for further experimentation.

### 2.5. Cell Transfection

Mouse EECs were inoculated in 6-well plates at 3 × 10^5^ cells per well. When the cells reached 80%, they were transfected with Opti-MEM diluted transfection complexes. Overexpression complexes consisted of pcDNA3.1(+)-PRAP1 (GenePharma, Shanghai, China) mixed with Hieff trans-liposome transfection reagent (Yeasen Biotech, Shanghai, China). The empty plasmid pcDNA3.1(+) was used as negative controls. After 6 h of cell transfection, the culture medium was replaced with a complete medium to continue culturing. After 48 h, the cells were collected and stored at −80 °C.

### 2.6. Embryo Culture and Embryo Adhesion Assay

The 4-cell stage embryos from mice on E 2 were cultured in KSOM medium. The embryos were divided into four groups receiving concentrations of 0 μg/mL, 6.25 μg/mL, 12.5 μg/mL, and 25 μg/mL PRAP1, respectively. The blastocyst formation rate was assessed 36 h post-treatment. In the embryo adhesion assay, blastocysts with zona pellucida removed were co-cultured with EEC overexpressing *Prap1* in a 24-well culture plate, with 4–5 blastocysts per well. After co-culture for 24 h, embryo adhesion was evaluated using a thermostatic oscillator set at 37 °C and 100 rpm for 30 s.

### 2.7. RNA Extraction and Quantitative Real-Time PCR (qRT-PCR)

Total RNA was extracted using the RNeasy Plus Mini Kit (Qiagen, NRW, Germany), and RNA purity was detected by Nanodrop. Total RNA was reverse transcribed using the PrimeScript Reverse Transcriptase Kit (TAKARA, Kusatsu, Japan). qRT-PCR was performed on a QuantStudio™ Real-Time PCR System using the Forget-Me-Not™ EvaGreen^®^ qPCR Master Mix kit (BIOTIUM, Fremont, USA), with each sample having at least three replicates. The qRT-PCR reaction system and reaction conditions are shown in Supplemental Tables S1 and S2. The data were analyzed and normalized to *Gapdh* mRNA levels by the 2^−ΔΔCt^ method. The primer sequences are shown in Table 1.

### 2.8. RNA Sequencing and Analysis

RNA was extracted from cells using standard methods. The quality of the RNA was tested using the Agilent 2100 bioanalyzer (Agilent Technologies Inc., California, USA). After the quality control test was passed, mRNA with polyA tails was enriched using Oligo(dT) magnetic beads. The obtained mRNA was randomly interrupted with divalent cations in the NEB Fragmentation Buffer. The library was constructed according to NEB library construction guidelines. The library preparations were sequenced on an Illumina Novaseq platform and 150 bp paired-end reads were generated. The read count data obtained from the gene expression level analysis of the samples was analyzed using DEseq2 software (Version: DEseq2 1.42.1). We used OmicStudio tools (https://www.omicstudio.cn/tool, accessed on 22 June 2024) to perform principal component analysis (PCA) and cluster analysis to generate heat maps and volcano maps. Differential gene screening between groups was conducted using the conditions *p* < 0.05 and fold change > 1.5. The differentially expressed genes (DEGs) were subjected to Gene Ontology (GO), Kyoto Encyclopedia of Genes and Genomes (KEGG), reactome enrichment analysis, and gene set enrichment analysis (GSEA). An enrichment plot was performed using the OmicStudio tools at https://www.omicstudio.cn/tool (accessed on 22 June 2024). The online tool YinOYang-1.2 (https://services.healthtech.dtu.dk/services/YinOYang-1.2, accessed on 26 November 2024) was used to predict mouse *Muc1* glycosylation sites, and String (https://cn.string-db.org, accessed on 27 November 2024) was used to construct a protein–protein interaction network.

### 2.9. Statistical Analysis

All experiments in this study were independently repeated at least three times, and GraphPad Prism 9.3 was used for statistical analysis. The data were expressed as the Means ± SEM. A one-way analysis of variance and a *t*-test were used for the data. At least a *p* < 0.05 was considered a significant difference.

## 3. Results

### 3.1. Estrogen Induces the Expression of Prap1 in Mouse Uterus

**Figure 1:**
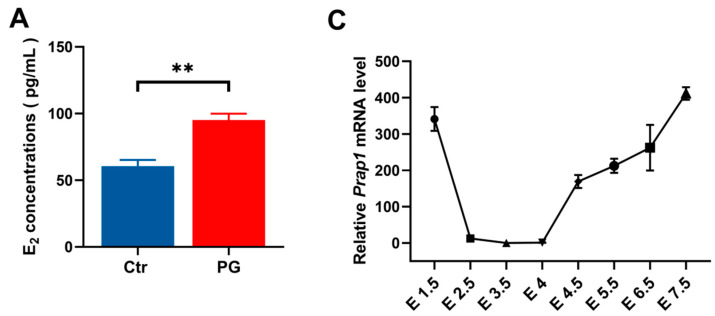

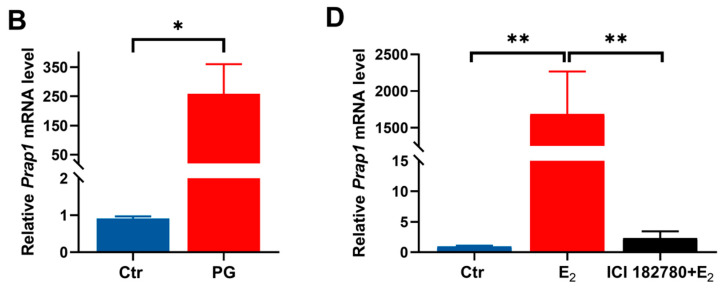
*Prap1* mRNA expression in uterus is regulated by estrogen. (**A**) PMSG combined with GnRH treatment increased estrogen levels in mice on E 3.5. *n* = 9. (**B**) PMSG combined with GnRH treatment increased *Prap1* mRNA expression in mice uteri on E 3.5. *n* = 3. (**C**) *Prap1* mRNA expression in mice uteri from E 1.5 to E 7.5. *n* = 3. (**D**) Estrogen-regulated *Prap1* mRNA expression in ovariectomy mice model. *n* = 3. *Prap1*, proline-rich acidic protein 1. Ctr, Control. PG, PMSG combined with GnRH treatment. E_2_, estradiol. ICI 182780, Estrogen receptor inhibitor. The presence of a vaginal plug was recorded as embryonal day 0.5 (E 0.5). * *p* < 0.05. ** *p* < 0.01.

This study used PMSG + GnRH-treated (PG) mice as a high estrogen level model. The results showed that the PG group had significantly higher estrogen levels than the control group on E 3.5 (Figure 1A). It was discovered that *Prap1* mRNA expression was significantly higher in the uterus of the PG group than in the control group on E 3.5 (Figure 1B). Next, the expression pattern of *Prap1* mRNA in the mouse uterus from E 1.5 to E 7.5 of the early pregnancy stage was analyzed. The results indicated that *Prap1* exhibited high expression in the uterus on E 1.5 and sharply decreased on E 2.5. Subsequently, its expression significantly decreased or became nearly undetectable on E 3.5 and E 4 compared to E 2.5. However, there was a notable surge in expression on E 4.5 following embryo implantation. As decidualization progressed, *Prap1* mRNA expression gradually increased in the uterus (Figure 1C). These results suggest that decreased Prap1 expression may be necessary during the implantation window and that estrogen may regulate *Prap1* to affect embryo implantation. To verify the regulatory relationship between estrogen and *Prap1*, subcutaneous injections of estrogen (E_2_) or an estrogen antagonist (ICI 182780) were administered to ovariectomized mice. The results showed a significant increase in *Prap1* mRNA expression in the uterus of mice treated with E_2_ for 24 h compared to the control group (Figure 1D). In the ICI 182780 + E_2_ group, the *Prap1* mRNA expression was significantly decreased compared to the E_2_ group (Figure 1D). The results suggested that estrogen could regulate *Prap1* expression, and high estrogen levels increased *Prap1* expression.

### 3.2. PRAP1 Impairs Embryo Implantation

**Figure 2:**
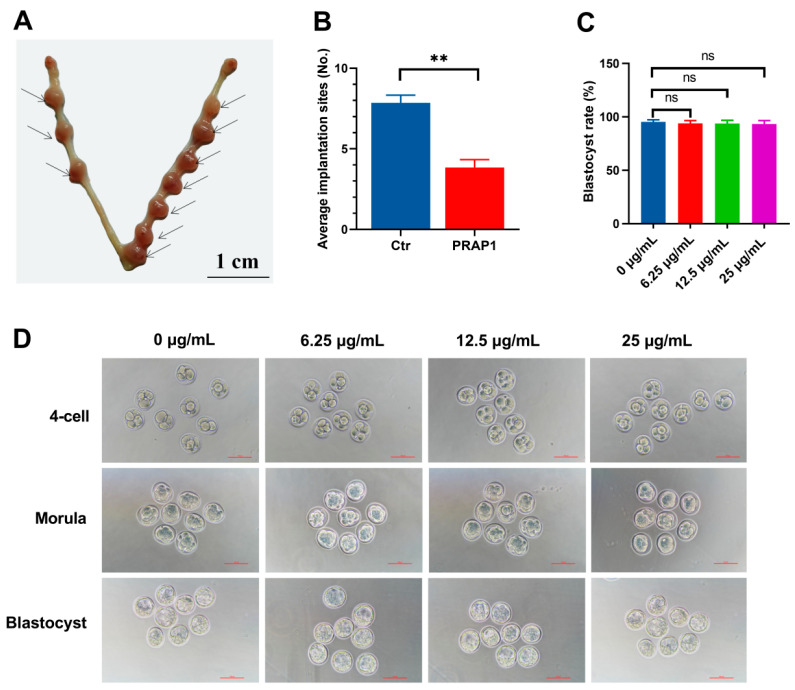
PRAP1 decreased embryo implantation but did not affect embryo development. (**A**,**B**) Uterine infusion of PRAP1 in mice reduced the number of embryo implantation sites on E 7.5. *n* = 8. The presence of a vaginal plug was recorded as embryonal day 0.5 (E 0.5). Scale bar: 1 cm. (**C**,**D**) Exogenous addition of PRAP1 protein to cultured mouse embryos did not affect early embryonic development. *n* = 5. Scale bar: 100 µm. *Prap1*, proline-rich acidic protein 1. Ctr, Control. ** *p* < 0.01. ns, no significance.

The study showed a decreased *Prap1* expression in the uterus on E 2.5 to E 3.5, so perfusion of PRAP1 protein into the uterine horn of mice on E 2.5 was performed. Perfusing PRAP1 into the uterus significantly decreased implantation sites on E 7.5 compared with the control group (Figure 2A,B). The result indicated that increased PRAP1 decreased the implantation rate.

Additionally, to exclude the effect of PRAP1 on early embryonic development, an in vitro embryo development test was conducted. The results revealed that the three groups of embryos treated with 6.25 μg/mL, 12.5 μg/mL, and 25 μg/mL PRAP1 successfully developed into blastocysts, with no significant differences in the blastocyst development rate compared to 0 μg/mL (Figure 2C,D). The results indicated that PRAP1 did not adversely affect early embryonic development but impaired embryo implantation.

### 3.3. Overexpression of Prap1 in Endometrial Epithelial Cells Altered Gene Expression Patterns

**Figure 3:**
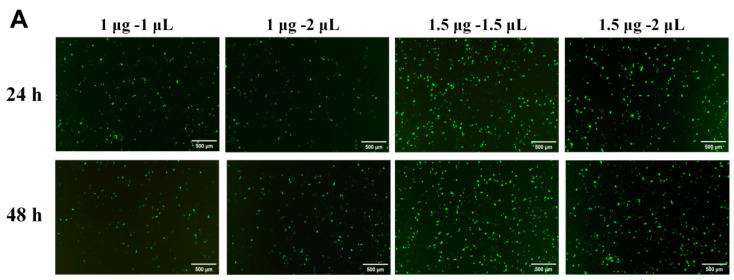

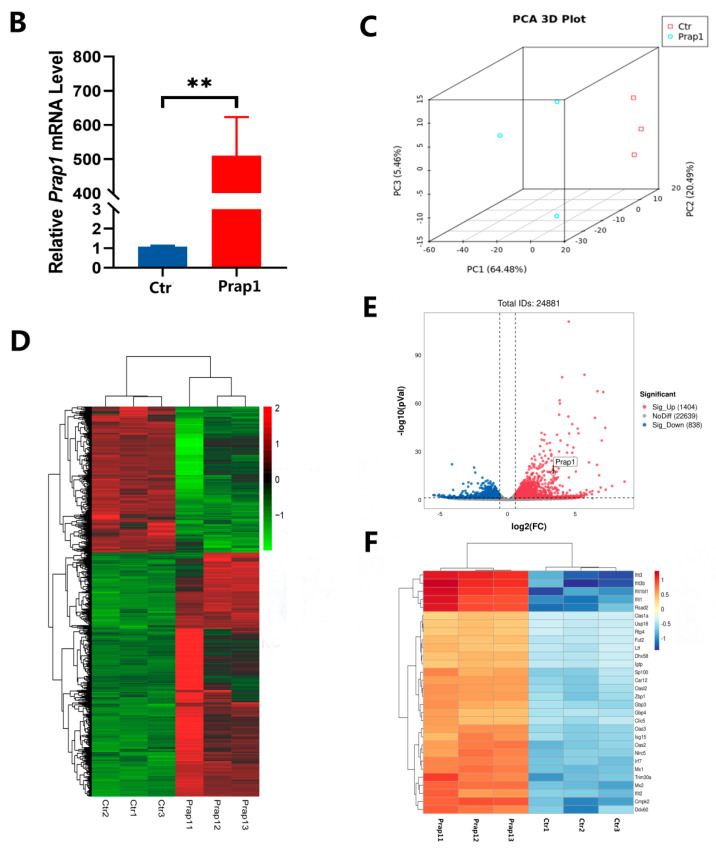
Overexpression of *Prap1* in endometrial epithelial cells altered the gene expression profile. (**A**) Optimization of transfection system in mouse endometrial cells. Scale bar: 500 µm. (**B**) Overexpression of *Prap1* significantly increased *Prap1* mRNA expression in endometrial epithelial cells. n = 4. (**C**) Endometrial epithelial cells with *Prap1* overexpression and Ctr cells were divided into two clusters by PCA. n = 3. (**D**) Heatmap of differentially expressed genes (DEGs) between *Prap1* overexpression group and Ctr group. n = 3. (**E**) Volcano plot of DEGs between *Prap1* overexpression group and Ctr group. n = 3. (**F**) Heatmap of Top30 DEGs. n = 3. *Prap1*, proline-rich acidic protein 1. Ctr, Control. ** *p* < 0.01.

We successfully overexpressed *Prap1* in primary mouse EECs (Figure 3A,B). Subsequently, transcriptome sequencing was performed on EECs overexpressing *Prap1* to investigate the *Prap1* regulatory mechanism. We performed principal component analysis (PCA) on the samples. The results showed that although the *Prap1* group was more discrete than the Ctr group, the Ctr and Prap1 groups were clearly divided into two clusters (Figure 3C). We performed a cluster analysis of differentially expressed genes (DEGs). The heatmap showed different gene expression patterns between the Ctr and Prap1 groups (Figure 3D). A total of 2242 DEGs were identified between the Ctr group and the *Prpa1* group, with 1404 upregulated and 838 down-regulated (Figure 3D). The transcriptome data revealed that *Prap1* mRNA abundance in the overexpression group was significantly higher than in the control group (Figure 3E). Here, we present the top 30 DEGs. Interestingly, the top 30 DEGs were increased in the *Prap1* group, including seven interferon-related genes (Figure 3F). Unexpectedly, all of these genes were upregulated in EECs after *Prap1* overexpression. In conclusion, our results demonstrated that overexpression of *Prap1* in mouse EECs alters gene expression patterns.

### 3.4. Overexpression of Prap1 Changed the Extracellular Matrix-Related Gene Expression in EECs

**Figure 4:**
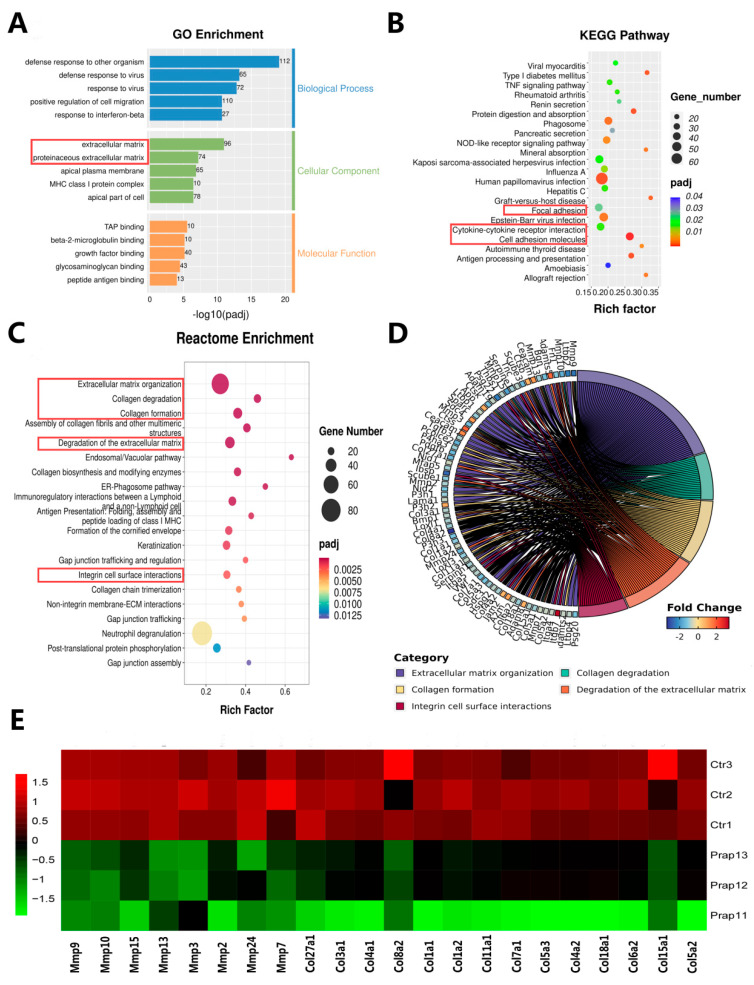
Transcriptome data revealed that overexpression of *Prap1* altered extracellular matrix-related adhesion in endometrial epithelial cells. (**A**) GO enrichment analysis of differentially expressed genes. (**B**) KEGG pathways enrichment analysis of differentially expressed genes. (**C**) Reactome enrichment analysis of differentially expressed genes. (**D**) Chord diagram of the 5 pathways related to extracellular matrix and collagen. (**E**) Heatmap of the 5 pathways related to extracellular matrix and collagen enriched in 8 Matrix metalloproteinases (Mmps) genes and 14 related-collagen genes. n = 3. *Prap1*, proline-rich acidic protein 1.

To further analyze the differences caused by the overexpression of *Prap1* in EECs, we performed an enrichment analysis of DEGs. We performed GO enrichment analysis for all DEGs. Biological processes were mainly enriched in defense response and cell migration. Cellular components were mainly enriched in extracellular matrix and the apical part of the cell. Molecular functions were mainly enriched in molecule binding (Figure 4A). Among the KEGG-enriched pathways, we found three adhesion-related pathways: focal adhesion, cytokine–cytokine receptor interaction, and cell adhesion molecules (Figure 4B). Reactome enrichment analysis showed that the DEGs were enriched in extracellular matrix and collagen (Figure 4C). Specifically, analysis of five collagen-related pathways revealed 71 genes, including eight matrix metalloproteinase-related genes and 14 collagen-related genes (Figure 4D). We analyzed the expression of these genes and showed that matrix metalloproteinase-related genes and collagen-related genes were significantly upregulated in the *Prap1* group compared with the Ctr group (Figure 4E). These gene changes may be detrimental to the establishment of endometrial receptivity, leading to the failure of embryo adhesion to EECs.

### 3.5. Overexpression of Prap1 Affected Embryo Adhesion by Increasing Muc1 Expression

**Figure 5:**
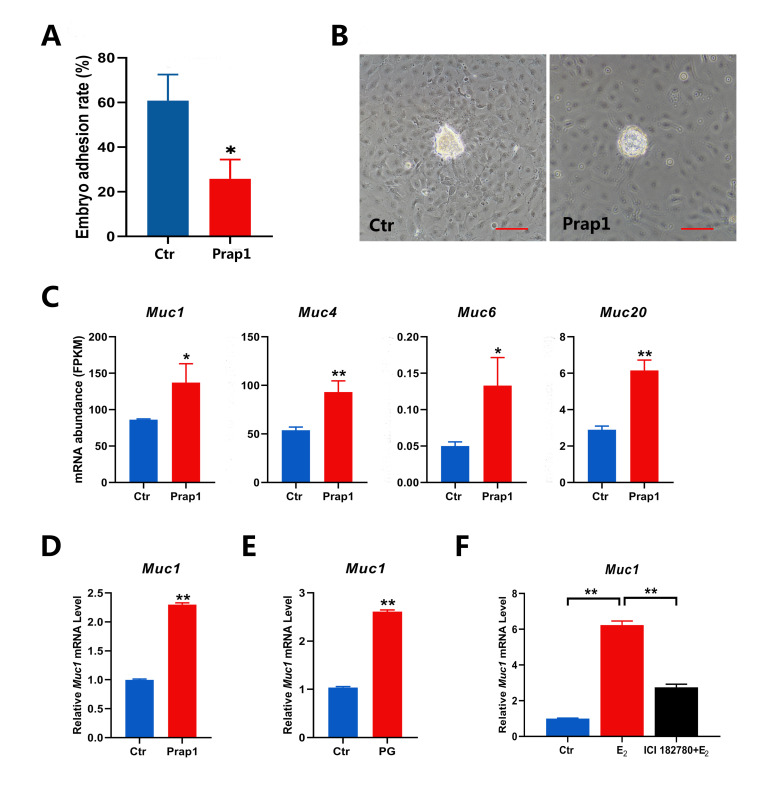
Overexpression of *Prap1* decreased embryo adhesion by increasing *Muc1* expression in endometrial epithelial cells. (**A**,**B**) Overexpression of *Prap1* decreased embryo adhesion in endometrial epithelial cells. n = 5. Scale bar: 100 µm. (**C**) Overexpression of *Prap1* increased the abundance of four mucin mRNAs in endometrial epithelial cells. n = 3. (**D**) Overexpression of *Prap1* increased *Muc1* mRNA expression in endometrial epithelial cells by qRT-PCR. n = 3. (**E**) *Muc1* expression is increased in the uterus on E 3.5 of a high estrogen level mouse model. n = 3. (**F**) Estrogen-regulated *Muc1* mRNA expression in ovariectomy mice model. n = 3. *Prap1*, proline-rich acidic protein 1. *Muc1*, mucin1. *Muc4*, mucin4. *Muc6*, mucin6. *Muc20*, mucin20. Ctr, Control. PG, PMSG combined with GnRH treatment. * *p* < 0.05. ** *p* < 0.01.

Based on the results of the previous experiments, we performed embryo adhesion tests in vitro. Overexpression of *Prap1* in EECs reduced embryo adhesion rates compared to control cells (Figure 5A,B). The time-dependent expression of mucins in EECs during implantation is critical for embryo adhesion. Four mucin family members were identified in the DEGs. The expression of these four mucins was significantly increased in the *Prap1* group compared with the Ctr group (Figure 5C). We focused on *Muc1* expression because its loss at the implantation site is critical for embryo adhesion. *Muc1* was also the most abundant expression among the four mucin family members. *Muc1* expression was examined in EECs overexpressing *Prap1*. *Muc1* mRNA expression was significantly upregulated in the *Prap1* group compared with the Ctr group by qRT-PCR, consistent with the transcriptome data (Figure 5D). Our previous experiments have shown that superovulation leads to a decrease in the number of implantation sites in mice. We examined *Muc1* expression in the uterus of mice on E 3.5 after superovulation. *Muc1* expression was significantly higher in the uterus of superovulatory mice than in normal pregnancies (Figure 5E). We used an ovariectomized mouse model to examine whether *Muc1* is regulated by estrogen. The results showed that estrogen injection significantly increased *Muc1* expression in the uterus of mice. However, this effect was lost after injection of an estrogen receptor antagonist (ICI 182780). This is consistent with the regulation of *Prap1* by E_2_. In short, the results suggested that estrogen promotes *Muc1* expression in EECs by increasing *Prap1*, leading to the failure of embryo adhesion.

### 3.6. Overexpression of Prap1 Activated Mucin-Type O-Glycosylation Biosynthesis

**Figure 6:**
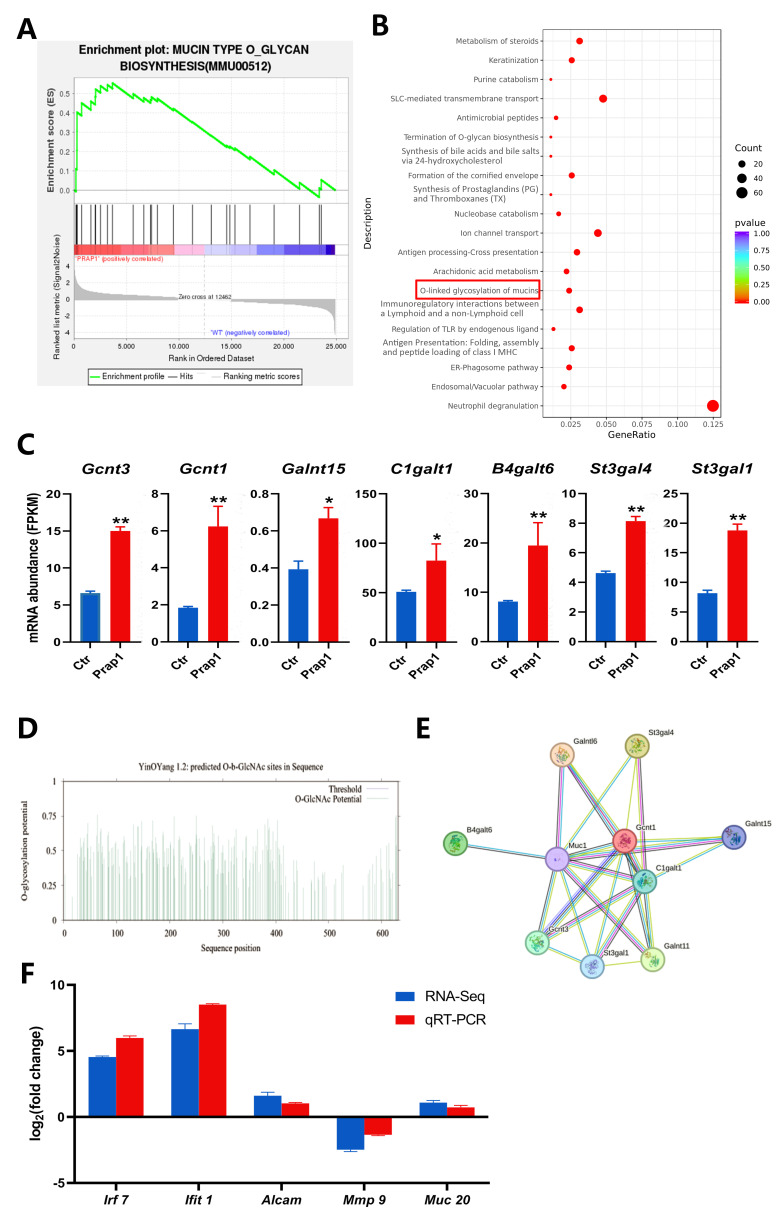
Overexpression of *Prap1* activated mucin O-glycosylation in endometrial epithelial cells. (**A**) GSEA enrichment analysis enriched in mucin type O-glycan biosynthesis. (**B**) KEGG pathways enrichment analysis of upregulated differentially expressed genes in overexpressing *Prap1* endometrial epithelial cells. (**C**) Overexpression of *Prap1* increased mucin type o-glycan biosynthesis gene abundance. n = 3. (**D**) MUC1 O-glycosylation site prediction. (**E**) Protein–protein interaction network diagram of o-glycosylation biosynthesis genes and *Muc1.* (**F**) The mRNA expression level of candidate genes was ascertained by RNA sequencing (RNA-Seq) and quantitative real-time PCR (qRT-PCR). * *p* < 0.05. ** *p* < 0.01.

In the uterus, *Muc1* is expressed on the surface of EECs. The extensive O-glycosylation of *Muc1* lubricates the cell surface and plays an important role in the cellular immune barrier. By performing GSEA analysis of all genes identified by transcriptome sequencing, mucin-type O-glycan biosynthesis was significantly enriched in EECs overexpressing *Prap1* (Figure 6A). In DEG enrichment analysis, O-O-linked glycosylation of mucins was also significantly enriched among pathways upregulated in EECs overexpressing *Prap1* (Figure 6B). We further analyzed the glycosylation-related gene expression. The results showed that key glycosylation genes were significantly upregulated in the *Prap1* group compared with the Ctr group (Figure 6C). We analyzed potential glycosylation sites for mouse *Muc1* and found 93 potential O-glycosylation sites (Figure 6D, Supplemental Table S3). Further analysis of the PPI network of *Muc1* and glycosylation-related genes revealed that *Muc1* interacted with glycosylation genes (Figure 6E). The results suggested that *Prap1* may lead to the failure of embryo adhesion to EECs by promoting O-glycosylation of *Muc1.*

### 3.7. RNA Sequencing Data Verification

*Irf7*, *Ifit1*, *Alcam*, *Mmp9*, and *Muc20* were selected as candidate genes based on the identified differential gene expression abundance and enrichment analysis results. These genes are mainly associated with immune response, extracellular matrix remodeling, and epithelial cell adhesion. These factors are essential for the establishment of uterine receptivity. The mRNA expression levels of candidate genes were detected by qRT-PCR. All the candidate genes of qRT-PCR results with RNA-seq results showed the same trend, which proved that the sequencing results were accurate and reliable (Figure 6F).

## 4. Discussion

Estrogen is critical in establishing uterine receptivity [23,24]. Abnormally high estrogen level was commonly caused by controlled ovarian hyperstimulation in humans and other mammals. High estrogen levels were detrimental to pregnancy establishment. In our previous study, estrogen level was significantly increased in mice by PMSG combined with GnRH treatment, resulting in embryo implantation failure [12]. PRAP1 is a secreted protein by specific epithelial cells and is crucial in maintaining epithelial cell homeostasis. *Prap1* was expressed and secreted in mice at estrus and down-regulated in the pre-implantation E 3.5 uterus but is significantly upregulated after implantation [22]. Similarly, our result found that *Prap1* expression was sharply decreased on E 2.5 compared with E 1.5. *Prap1* expression almost disappeared on E 3.5 and E 4, but gradually increased with decidualization progression from E 4.5. This study showed that the absence of *Prap1* is crucial for successful implantation during the implantation window. The expression pattern of *Prap1* was similar to the changes in estrogen throughout the pre-implantation. Further, a high estrogen level mouse model was created using PMSG combined with GnRH protocol. This model demonstrated a significant increase in *Prap1* mRNA levels in the mice uteri on E 3.5. There was a significant decrease in the number of embryo implantation sites in the uterus of mice treated with this protocol. However, the excessively high expression of *Prap1* may contribute to embryo implantation failure under high estrogen levels.

Previous studies have reported that *Prap1* is a differentially expressed gene caused by altered estrogen levels [12]. Further experiments using an ovariectomized mouse model found a significant increase in *Prap1* mRNA level in the uterus following exogenous estrogen administration, and treatment with ICI 182780 before estrogen injection blocked the estrogen-induced increase in *Prap1* mRNA level. Another study found that the PRAP1 expression in the endometrium is inversely related to the class I histone deacetylase (IHDAC) expression. Interestingly, the IHDACs inhibitor (sodium butyrate) can upregulate PRAP1 expression, while an estrogen receptor antagonist (ICI 182780) can down regulate PRAP1 expression by increasing IHDACs levels [13]. The results showed that estrogen regulates *Prap1* expression through estrogen receptors.

Moreover, the PRAP1 protein-infused experiment showed that the number of embryo implantation sites significantly decreased after uterine infusion of PRAP1 in mice on E 2.5. However, a study showed that the number of implanted embryos was reduced on E 7.5 when female mice were injected with anti-PRAP1 on E 2.5 [13]. The result suggests that the reduction of PRAP1 is detrimental to maintaining pregnancy. In summary, the time-specific regular expression pattern of *Prap1* is essential for embryo implantation, and increasing *Prap1* in the mouse uterus during implantation can result in impaired embryo implantation. Embryo implantation needs a receptive uterus and an embryo capable of implantation. On E 2.5–E 3.5, exposure to perfused PRAP1 after the embryo enters the uterus may impact embryo development and result in implantation failure. This study utilized PRAP1 to culture 4-cell embryos and develop them to the blastocyst stage in vitro. The findings indicated that PRAP1 did not affect embryonic development.

During embryo implantation, the blastocyst undergoes localization, adhesion, and invasion after hatching to establish stable contact with the mother [25]. The EEC is the first cell type that the embryo comes into contact with in the uterus. Embryo adhesion to the endometrial epithelial cells is critical in embryo implantation [26,27,28]. Studies have shown that PRAP1 is specifically expressed in uterine EECs [13,29]. We explored the role of *Prap1* in EECs using an in vitro cell model. Transcriptome sequencing was performed on EECs overexpressing *Prap1* to analyze the Prap1-induced differences in these cells comprehensively. Compared to normal cells, EECs overexpressing *Prap1* showed a different gene expression profile. We identified a total of 2242 differentially expressed genes (DEGs).

Most of the top 30 DEGs were related to immunity, including seven interferon-related genes. The immune microenvironment of the uterus is crucial for embryo implantation. The significant changes of these genes may affect the regulation of the immune microenvironment of the uterus, thereby affecting embryo implantation. GO enrichment analysis was mainly enriched in cellular defense and extracellular components. Reactome enrichment analysis also enriched many extracellular matrix pathways related to collagen, which were enriched in many of the same genes. These genes may be key genes in the regulation of the extracellular matrix. Endometrial remodeling is a crucial step in embryo implantation, which involves remodeling and degradation of the extracellular matrix [30,31]. Integrin-mediated recognition and adhesion cells and extracellular matrix proteins, such as collagen, laminin, and fibronectin, are essential for embryo adhesion to the endometrium during implantation [32,33]. Several integrin and matrix metalloproteinase genes were enriched in five collagen-related pathways. *Prap1*-induced changes in these genes may harm embryonic adhesion to EECs. KEGG enrichment analysis also found three pathways related to cell adhesion: focal adhesion, cytokine–cytokine receptor interaction, and cell adhesion molecules. Overall, the transcriptome results indicated that *Prap1* overexpression in EECs affected the extracellular matrix-related gene expression and signaling pathway, potentially reducing embryo adhesion.

We performed embryo adhesion assays in vitro. Overexpression of *Prap1* in EECs notably reduced the number of adhesive embryos. The time-dependent expression of mucins is critical for embryo implantation. We identified four mucin family members among the DEGs, including *Muc1* and *Muc20*, which are known to play important roles in embryo implantations [34,35]. We focused on *Muc1* expression because *Muc1* has been widely reported to be down-regulated during embryo implantation [36,37,38]. The increased expression of *Muc1* in EECs before implantation prevents embryo adhesion [39]. When the implantation window is opened, *Muc1* begins to decrease, the endometrial luminal epithelium comes into contact with the blastocyst, and adhesion occurs. Therefore, appropriate reduction of *Muc1* expression is crucial for uterine receptivity. The increase in *Muc1* on the cell surface can inhibit cell–cell adhesion [40]. Thus, dysregulation of *Muc1* expression at implantation may prevent embryo implantation and the establishment of early pregnancy failure. Overexpression of *Prap1* in EECs increased *Muc1* mRNA expression. Similarly, *Muc1* mRNA expression was significantly upregulated in the E 3.5 uterus of mice with a high estrogen model. Similar to the way estrogen regulates *Prap1*, *Muc1* was also regulated by estrogen, which is consistent with previous reports. These results suggested that estrogen can regulate *Muc1* expression through *Prap1*.

The endometrial luminal epithelium is covered with highly glycosylated mucins that regulate embryo adhesion during implantation [34]. O-linked glycosylation of the mucins pathway was enriched in both GSEA enrichment analysis and KEGG upregulated pathways. We analyzed the expression of glycosylation-related genes and found that this gene expression abundance was significantly upregulated in EECs overexpressing prap1. *Muc1* was present on the surface of normal epithelial cells and is a Ser/Thr/Pro-rich transmembrane protein with high O-glycosylation [41]. The extended O-glycan on *Muc1* forms a mucinous gel that maintains cell surfaces lubricated and hydrated, playing an important role in the immune barrier against microbial and proteolytic attacks [42]. This immune barrier may be detrimental to blastocyst adhesion in the uterus during implantation. We analyzed the protein sequence of MUC1 in mice and found 93 potential O-glycosylation sites. Moreover, we constructed the interaction network between *Muc1* and glycosylation key genes and found that *Muc1* was closely related to glycosylation key genes. Our results suggested that *Prap1* may regulate *Muc1* O-glycosylation to affect embryo adhesion, leading to implantation failure. Regrettably, our study did not achieve detection at the protein level. Additionally, further investigation is required to ascertain the glycosylation level and sites of MUC1 to identify key targets. These findings will form a crucial foundation for advancing our understanding and elucidation of estrogen’s regulatory role in embryo implantation.

## 5. Conclusions

In summary, the present study identifies the role of estrogen in regulating *Prap1* expression. It revealed that high estrogen levels led to the overexpression of *Prap1* in the uterus. On the one hand, *Prap1* increased *Muc1* expression in EECs, and on the other hand, it may promote *Muc1* O-glycosylation. These changes prevented the adhesion of embryos to EECs, leading to implantation failure. Our findings provide new insights into the regulation of embryo implantation by estrogen and provide a theoretical reference for pregnancy failure caused by high estrogen.

## Figures and Tables

**Table 1:** Primer sequences used for qRT–PCR.

Gene	Primer Sequences (5′-3′)	Accession No.
*Gapdh*	Forward: AGGTCGGTGTGAACGGATTTG	NM_001289726.2
	Reverse: TGTAGACCATGTAGTTGAGGTCA	
*Prap1*	Forward: AGAAGGTCTGGGATACTAGAGCC	NM_009475.2
	Reverse: GCATCTGGACGCTTTTCCTC	
*Muc1*	Forward: AGTGCCTCTGACGTGAAGTCAG	NM_013605.2
	Reverse: GGGAGGGAACTGCATCTCATTC	
*Irf7*	Forward: CCTCTGCTTTCTAGTGATGCCG	NM_016850.3
	Reverse: CGTAAACACGGTCTTGCTCCTG	
*Ifit1*	Forward: TACAGGCTGGAGTGTGCTGAGA	NM_008331.4
	Reverse: CTCCACTTTCAGAGCCTTCGCA	
*Alcam*	Forward: AGGAACATGGCGGCTTCAACGA	NM_009655.3
	Reverse: ACACCACAGTCGCGTTCCTACT	
*Mmp9*	Forward: GCTGACTACGATAAGGACGGCA	NM_013599.5
	Reverse: TAGTGGTGCAGGCAGAGTAGGA	
*Muc20*	Forward: ATGGAGGCTTCCTCCTTGTACG	NM_001145874.1
	Reverse: ACCAGATGGCTCGTGAGTCACT	

## Data Availability

The datasets generated during the study are available from SAR (https://www.ncbi.nlm.nih.gov/bioproject/PRJNA1122317, accessed on 11 June 2024.) or the authors.

## Supplementary Materials

The following supporting information can be downloaded at: https://www.mdpi.com/article/10.3390/biom15060852/s1, Table S1: System of qRT-PCR reaction; Table S2: The reaction conditions of qRT-PCR; Table S3: MUC1 O-glycosylation site prediction.
