# Supplementary material for: Estrogen-Regulated Proline-Rich Acidic Protein 1 in Endometrial Epithelial Cells Affects Embryo Implantation by Regulating Mucin 1 in Mice
Source: Biomolecules. 2025 Jun 11;15(6):852. doi: 10.3390/biom15060852 (PMC12190424; doi:10.3390/biom15060852)
Supplement: Supplementary file 1 [file biomolecules-15-00852-s001.zip › biomolecules-3609978.pdf]

Supplemental Table S1 System of qRT-PCR reaction

Reagent	Volume (μL)
2x qPCR Master Mix	10
Forward Primer	0.4
Reverse Primer	0.4
cDNA	1
ROX	3
Rnase Free H_2O	5.2
Total Volume	20

Supplemental Table S2 The reaction conditions of qRT-PCR

Steps	Temperature	Time	Cycles
pre-denaturation	95 °C	2 min	1
denaturation	95 °C	5 s	40
annealing	60 °C	30 s	

Supplemental Table S3 MUC1 O-glycosylation site prediction.

SeqName	Residue		O-GlcNAc	Potential
Sequence	32	T	+++	0.5917
Sequence	33	S	+++	0.519
Sequence	34	S	+++	0.5363
Sequence	38	S	+++	0.5612
Sequence	39	S	+++	0.5924
Sequence	44	T	+++	0.6214
Sequence	45	T	+++	0.5202
Sequence	46	T	+++	0.699
Sequence	51	S	+++	0.5327
Sequence	63	S	+++	0.6081
Sequence	64	T	++++	0.7596
Sequence	66	S	+++	0.5536
Sequence	70	S	+++	0.6384
Sequence	71	S	+++	0.6597
Sequence	73	S	+++	0.5322
Sequence	83	S	+++	0.6107
Sequence	84	T	+++	0.6363
Sequence	90	S	+++	0.5184
Sequence	93	S	+++	0.5242
Sequence	97	T	+++	0.5504
Sequence	98	T	+++	0.5649
Sequence	103	S	+++	0.5114
Sequence	113	T	+++	0.5569
Sequence	114	S	+++	0.6056
Sequence	115	S	+++	0.5737
Sequence	126	S	+++	0.5016
Sequence	132	S	+++	0.6886
Sequence	135	S	+++	0.5381
Sequence	140	T	+++	0.6744
Sequence	141	T	+++	0.6761
Sequence	146	S	+++	0.5013
Sequence	147	T	+++	0.7111
Sequence	148	S	+++	0.5319
Sequence	157	S	+++	0.5182
Sequence	158	S	+++	0.531
Sequence	161	T	+++	0.5336
Sequence	167	S	+++	0.571
Sequence	168	T	+++	0.7183
Sequence	170	S	+++	0.5123
Sequence	174	S	+++	0.5969
Sequence	175	S	+++	0.5514
Sequence	187	S	+++	0.6107
Sequence	188	T	+++	0.6363

Sequence	194	S	+++	0.5184
Sequence	197	S	+++	0.5176
Sequence	201	T	+++	0.5416
Sequence	202	T	+++	0.5647
Sequence	207	S	+++	0.5136
Sequence	208	T	+++	0.7458
Sequence	217	T	+++	0.5757
Sequence	218	S	+++	0.6054
Sequence	219	S	+++	0.584
Sequence	238	T	+++	0.5584
Sequence	239	S	+++	0.5287
Sequence	240	S	+++	0.5388
Sequence	249	S	+++	0.5478
Sequence	250	T	++++	0.7509
Sequence	251	S	+++	0.6162
Sequence	252	S	+++	0.5859
Sequence	256	S	+++	0.5894
Sequence	266	T	+++	0.5973
Sequence	278	T	+++	0.5328
Sequence	281	T	+++	0.6816
Sequence	283	T	+++	0.5597
Sequence	284	S	+++	0.6251
Sequence	285	S	+++	0.5393
Sequence	292	S	+++	0.6076
Sequence	301	T	+++	0.5389
Sequence	312	T	+++	0.5798
Sequence	317	S	+++	0.5239
Sequence	322	T	+++	0.5319
Sequence	323	T	+++	0.5961
Sequence	326	T	+++	0.6556
Sequence	338	S	+++	0.5022
Sequence	344	S	+++	0.5966
Sequence	345	S	+++	0.6069
Sequence	357	T	+++	0.5304
Sequence	363	T	+++	0.5339
Sequence	366	S	+++	0.6531
Sequence	375	S	+++	0.5044
Sequence	380	S	+++	0.6889
Sequence	382	T	+++	0.5408
Sequence	385	T	+++	0.5043
Sequence	386	T	+++	0.7283
Sequence	387	S	+++	0.6496
Sequence	388	S	+++	0.6684
Sequence	391	T	+++	0.5094
Sequence	395	S	+++	0.6388
Sequence	400	T	+++	0.5691
Sequence	404	S	+++	0.7196
Sequence	405	T	+++	0.5209

Sequence	407	S	+++	0.5112
Sequence	415	S	+++	0.5392

Note: Potential values > 0.5 were considered potential glycosylation sites.
